# Evaluation of orthodontic mini-implant placement: a CBCT study

**DOI:** 10.1186/s40510-014-0061-x

**Published:** 2014-11-18

**Authors:** Shilpa Kalra, Tulika Tripathi, Priyank Rai, Anup Kanase

**Affiliations:** Department of Orthodontics and Dentofacial Orthopaedics, Maulana Azad Institute of Dental Sciences, New Delhi, 110002 India

**Keywords:** CBCT, Digital RVG, Mini-implant

## Abstract

**Background:**

Optimal positioning of orthodontic mini-implants is essential for a successful treatment with skeletal anchorage. This study aims to compare the accuracy of two-dimensional radiographs with a cone beam computed tomography (CBCT) for mini-implant placement.

**Methods:**

An ideal site for mini-implant placement at the buccal interradicular space between the second premolar and the first molar was determined for 40 sites (in 13 patients aged 14 to 28 years) by using CBCT data. The mini-implant placement procedure was then divided into two groups. In CBCT group, mini-implants were placed at the sites determined from CBCT data. In RVG group, mini-implants were placed with the help of two-dimensional digital radiographs and a custom made guide. Postplacement CBCT scans were obtained to determine the accuracy of the mini-implant placement. The results were statistically analyzed with a Mann-Whitney test.

**Results:**

A statistically significant difference (*p* value = 0.02) was observed between the two groups for deviation from an ideal height of placement of the mini-implants. Deviations in mesiodistal positioning and angular deviation showed a statistically non-significant difference. Three out of twenty mini-implants in the RVG group showed root contact in the mandibular arch that may be attributed to the narrower interradicular space and reduced accessibility in the mandibular posterior region.

**Conclusions:**

Although CBCT provides an accurate three-dimensional visualization of the interradicular space, the two-dimensional intraoral radiograph of the interradicular area provides sufficient information for mini-implant placement. Considering the amount of radiation exposure and cost with the two techniques, it is recommended to use two-dimensional radiographs with a surgical guide for a routine mini-implant placement.

## Background

The use of orthodontic mini-implants as an absolute anchorage device has seen a marked increase in orthodontic treatment [1,2]. Despite their advantages over the extraoral anchorage methods, mini-implants can occasionally loosen during treatment and eventually fail to provide firm anchorage [3–5]. A study showed that the rates of mini-implant failure vary between 11% and 30% [6].

The two major factors that clinicians should consider for mini-implant placement are safety and stability. Safety is related to avoiding root damage during implant placement in the interradicular space. Stability, especially initial stability, which plays a major role in preventing premature loosening of mini-implants, is obtained by placing the mini-implants in the alveolar bone with sufficient quantity and quality [3,7].

Fayed et al [8] suggested that the optimal sites for mini-implant placement are between the second premolar and first molar and between the first and second molars at the buccal aspect of the posterior region of both jaws. Successful placement of mini-implants at the desired site is critical for their incorporation into the clinical practice. Kim et al [9] presented a surgical guide system that used cone beam computed tomography (CBCT) images to replicate the dental models. Surgical guides for the proper positioning of orthodontic mini-implants were fabricated on the replicas and used for precise placement. Liu et al [10] introduced a CAD/CAM template with preoperative simulation for orthodontic miniscrew placement. Similarly, other authors have reported the extensive use of three-dimensional planning with the help of surgical guides, stents, and templates for accurate mini-implant positioning [11,12]. Although CBCT provides additional diagnostic and therapeutic information, it exposes the patients to a higher level of radiations than the conventional radiographs [13–15]. Thus, there is a need to assess whether the use of such an expensive and laborious procedure is required or not.

However, most clinicians routinely place mini-implants without 3D planning and use the two-dimensional radiographs for presurgical treatment planning. In an effort to minimize insertion errors, several innovative surgical guides have been used which help in avoiding root injury and subsequent patient discomfort [16–20].

Despite the extensive use of two-dimensional radiographs for mini-implant placement, little has been recorded in literature to determine their accuracy for site selection and position of mini-implants thereafter.

The present study was aimed at evaluating the accuracy of two-dimensional radiographs for site selection and orthodontic mini-implant placement.

## Methods

The present study evaluated 40 mini-implant placement sites in 13 patients (10 females and 3 males) aged between 14 and 28 years from North Indian population group who reported for orthodontic treatment to the Department of Orthodontics, Maulana Azad Institute of Dental Sciences, Delhi. The included cases required the extraction of the upper and/or lower first premolars with high anchorage consideration and were represented by eight class I bidentoalveolar protrusion patients, four class II division 1 malocclusion patients, and one class III surgical case. Twenty-four mini-implants (1.5 × 9 mm Infinitas mini-implant system, DB Orthodontics Limited, West Yorkshire, UK) were placed in the maxillae and sixteen in the mandible. The study was sanctioned by the Institutional Ethics Committee and informed consent was obtained from all the patients/guardians of the patients. The patients who agreed to have CBCT scans prior to and after mini-implant placement were included in the study. Pre- and postplacement CBCT scans were taken at an interval of 3 months. Patients with mixed dentition, missing teeth, severe periodontitis, and systemic diseases contraindicating the procedure were excluded.

The study design was randomized block design in which the 40 sites (sample units) were first grouped as 20 pairs (left and right interradicular sites in the same arch in a patient as one pair). All the 20 pairs were then randomly allocated by using split mouth system into two groups such that mini-implant placement was guided by CBCT on one side and RVG (digital intraoral periapical radiograph) on the other side in all the patients.

All the patients were treated with a preadjusted edgewise appliance of MBT prescription (0.022 × 0.028-in. slot). A sequence of aligning wires was used for leveling and alignment until a 0.019 × 0.025-in. stainless steel arch wire could be passively engaged.

After initial leveling and alignment, CBCT scans ( iCAT Cone beam three-dimensional imaging system, Imaging Sciences, Hatfield, PA, USA) were performed for all the patients for the determination of the ideal site for mini-implant placement in the buccal interradicular space between the second premolar and the first molar in maxillary and/or mandibular arches (with arch wires in place). CBCT scan was taken with the dosimetry parameters of 120 kV, 37.07 mA, and 40 s scan time. Measurements were performed by an expert oral and maxillofacial radiologist using iCAT vision software for bone density assessment and linear measurements and Digimizer (Version 4.0, MedCalc Software, Ostend, Belgium) image analysis software for angular measurements.

### Determination of ideal site for mini-implant placement

**Figure 1:**
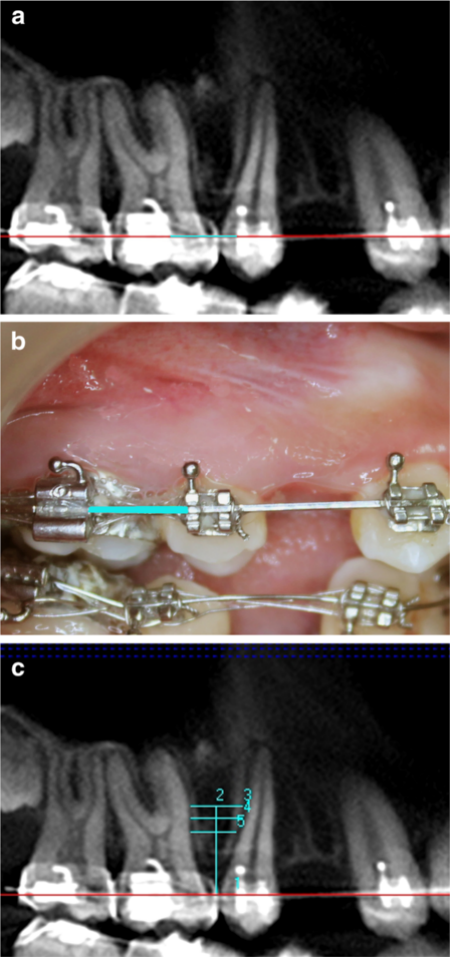
**Orthodontic arch wire in second premolar and first molar region taken as standard reference for measurements. (a)** Panorex View of CBCT image, **(b)** correlated clinically, and **(c)** various heights from the arch wire within the limits of mucogingival junction selected for bone measurements.

**Figure 2:**
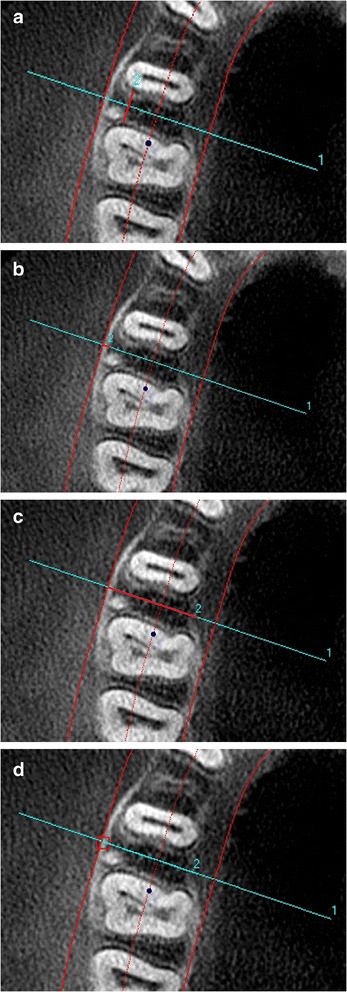
**Bone measurements. (a)** Mesiodistal distance,** (b)** buccal cortical bone thickness, **(c)** buccolingual thickness, and **(d)** bone density.

Orthodontic arch wire in the second premolar and the first molar region was taken as a reference for all the measurements (Figure 1a,b). Four bone measurements namely mesiodistal distance, cortical bone thickness, buccolingual thickness, and bone density were obtained in the axial slice sections corresponding to the various heights from the arch wire within the limits of mucogingival junction (Figure  1c and Figure 2). The level of mucogingival junction in the second premolar and the first molar region was predetermined clinically, and its distance from the arch wire was also calculated. Based on the measurements performed by an oral radiologist, the vertical level with a maximum mesiodistal interradicular distance with optimum buccal cortical plate thickness, bone density, and buccolingual thickness was considered to be the ideal height for mini-implant placement. Hence, ideal site for all the mini-implants were identified (center of the mesiodistal space at the determined ideal height).

However, this information was given to the operator only for CBCT-guided side during the mini-implant placement.

### CBCT group

In the CBCT group, the ideal sites as determined in the CBCT images were correlated clinically for the correct mesiodistal positioning of mini-implants at the desired height.

#### Determination of the point of entry of mini-implant clinically by using CBCT data

1. A perpendicular was dropped from the ideal point of insertion of mini-implant to the arch wire (in CBCT image) (Figure **3**a).

**Figure 3:**
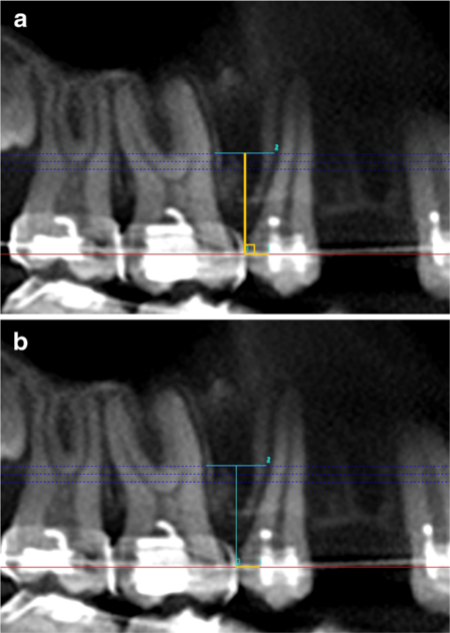
**Determination of the point of entry of mini-implant by using CBCT data. (a)** A perpendicular is dropped from ideal point of insertion of mini-implant to the arch wire. **(b)** Distance of point of intersection of perpendicular on the arch wire to distal end of second premolar bracket.
2. The distance of the point of intersection of the perpendicular on the arch wire to the distal end of the second premolar bracket was measured (in the CBCT image) (Figure **3**b).
  This distance was used clinically to mark the mesiodistal positioning of the mini-implant on the CBCT-guided side.
3. The ideal height for the mini-implant placement was marked intraorally as determined in the CBCT image.

The operator was trained by the oral and maxillofacial radiologist for interpreting the CBCT measurements. Based on the measurements derived, the operator inserted the mini-implants at the identified sites under local anesthesia.

### Digital intraoral radiography/digital RVG group

**Figure 4:**
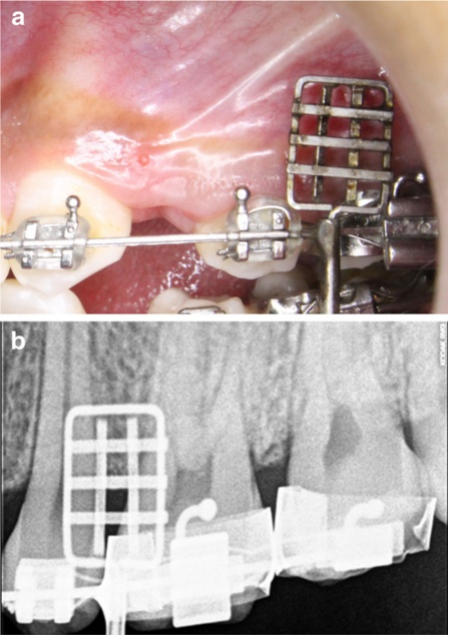
**Custom made guide in second premolar and the first molar region.** As seen **(a)** clinically and **(b)** radiographically.

An intraoral periapical view of the second premolar and the first molar region with custom made guide [21] in place using digital RVG (Kodak RVG 5100, Marne-la-vallée, France) was taken (Figure 4). The custom made guide consists of two parts: a grid and a grid holder. This grid provided an option of nine cells and the area which seemed to be centered between the adjacent roots and at an optimal height was identified as the site for mini-implant placement. A bleeding point was created with the explorer through the guide. The guide was then removed and mini-implant was inserted at the identified site under local anesthesia.

All the mini-implants were inserted by a single operator.

#### Postplacement CBCT

After insertion of the mini-implants, another CBCT scan for comparative evaluation of the accuracy of the site selection and mini-implant placement between the two groups was performed by an expert oral and maxillofacial radiologist.

The following parameters were observed in the postplacement CBCT.

**Figure 5:**
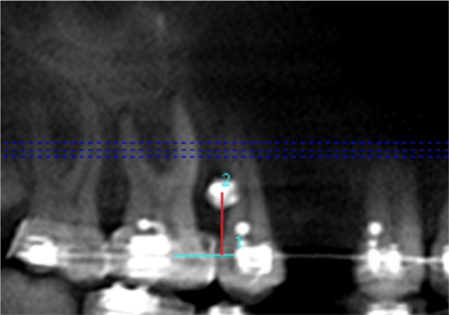
**Height of mini-implant from arch wire.**

1. Height of the mini-implant from the arch wire (Figure **5**)

  Deviation of height of mini-implant (DM_H_) from the ideal height was determined.
2. Mesiodistal position of the mini-implant at the interradicular area
  The ideal path of insertion of the mini-implant is drawn on the axial slice view and following parameters were measured.
    i. Deviation of the point of entry of mini-implant (DM_EP_) (Figure **6**a)

**Figure 6:**
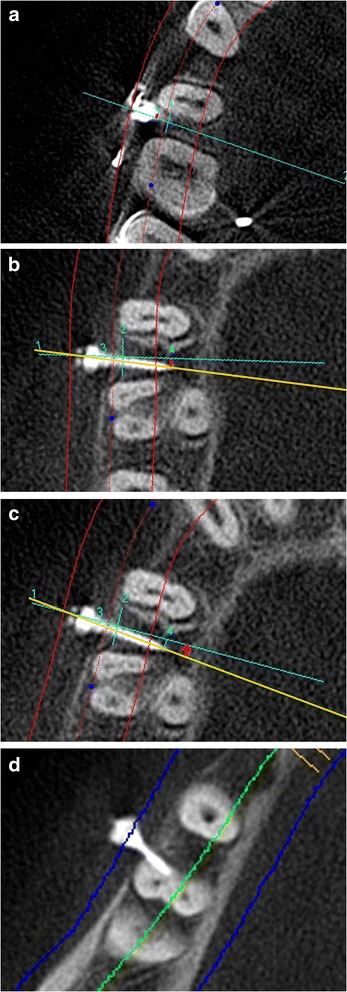
**The post insertion measurements. (a)** Deviation of the point of entry of the mini-implant (DM_EP_). **(b)** Deviation of the tip of mini-implant (DM_T_).** (c)** Angular deviation of path of mini-implant (DM_A_). **(d)** Root proximity to the mini-implant.
    ii. Deviation of the tip of mini-implant (DM_T_) (Figure **6**b)
    iii. Angular deviation of the mini-implant (DM_A_) from the ideal path (Figure **6**c)
3. Root contact of the mini-implant in the axial slice view (Figure **6**d)

### Statistical analysis

The means and standard deviations for DM_H_, DM_EP_, DM_T_, and DM_A_ were calculated for the mini-implants placed in both groups. The data was tested for normality and it showed a non-normal distribution. Hence, the comparison of the means of DM_H_, DM_EP_, DM_T_, and DM_A_ between the groups and within each group was done by independent samples Mann-Whitney *U* test (non-parametric test) with a *p* value of less than 0.05. Since the aim of the study was to compare between the two groups, only the absolute values without ‘+’ or ‘−’ signs were considered.

## Results

**Figure 7:**
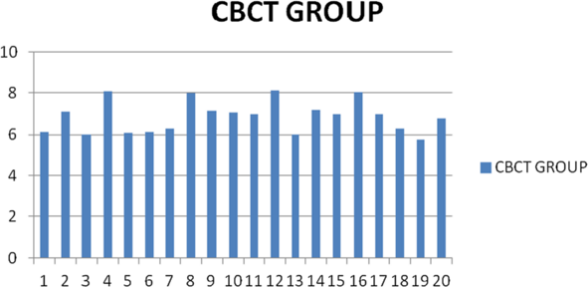
**Graphical representation of the height of mini-implants in CBCT group (in postplacement CBCT).**

**Figure 8:**
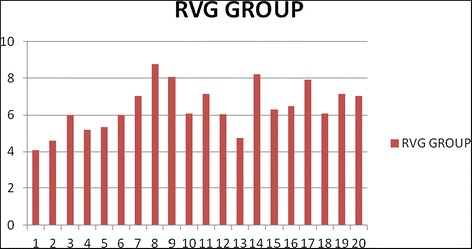
**Graphical representation of the height of mini-implants in RVG group (in postplacement CBCT).**

The heights at which mini-implants were placed in the CBCT group and RVG group are graphically represented in Figures 7 and 8, respectively, with an average height of mini-implants being 6.85 mm in CBCT group and 6.40 mm in RVG group. Mean values for DM_H_, DM_EP_, DM_T_, and DM_A_ for the CBCT group and RVG group along with the comparison of these parameters are tabulated in Table 1. Statistically significant difference (*p* value = 0.02) was observed between the two groups for mean DM_H_, with the deviation being 0.0985 mm in CBCT group and 0.565 mm in RVG group. Mini-implants were placed at a lower height (closer to the arch wire) in the RVG group. The differences in the mean values for DM_EP_, DM_T_, and DM_A_ were statistically non-significant.

**Table 1:** **Comparison of mean deviation of mini-implants from ideal position between CBCT group and RVG group with level of significance**

**Variable**	**CBCT group (** ***n*** **= 20)**	**RVG group (** ***n*** **= 20)**	**Significance (** ***p*** **value)**
Mean DM_H_ (in mm)	0.0985	0.565	0.02*
Mean DM_EP_ (in mm)	0.391	0.651	0.143
Mean DM_T_ (in mm)	0.586	1.038	0.204
Mean DM_A_ (in degrees)	4.7	6.2	0.624

*Significant difference in mean deviation of height of mini-implant (*p* < 0.05)

DM_H_, deviation of height of mini-implant; DM_EP_, deviation of point of entry of mini-implant; DM_T_, deviation of tip of mini-implant; DM_A_, angular deviation of mini-implant from the ideal path.

Intragroup comparisons are depicted in Tables 2 and 3. No statistically significant difference was observed on the right and left side and in the maxillary and mandibular arches in each of the groups.

**Table 2:** **Comparison of mean deviation of mini-implants from ideal position between right and left side in CBCT group and RVG group with level of significance**

**Variable**	**CBCT group**			**RVG group**		
	**Right side (** ***n*** **= 10)**	**Left side (** ***n*** **= 10)**	**Significance (** ***p*** **value)**	**Right side (** ***n*** **= 10)**	**Left side (** ***n*** **= 10)**	**Significance (** ***p*** **value)**
Mean DM_H_ (in mm)	0.091	0.106	0.939	0.768	0.362	0.226
Mean DM_EP_ (in mm)	0.361	0.422	0.662	0.57	0.733	0.702
Mean DM_T_ (in mm)	0.756	0.079	0.256	0.815	1.261	0.111
Mean DM_A_ (in degrees)	4.6	0.2	0.873	2.8	5.6	0.563

DM_H_, deviation of height of mini-implant; DM_EP_, deviation of point of entry of mini-implant; DM_T_, deviation of tip of mini-implant; DM_A_, angular deviation of mini-implant from the ideal path.

**Table 3:** **Comparison of mean deviation of mini-implants from ideal position between maxillary and mandibular sites in the CBCT group and RVG group, respectively, with level of significance**

**Variable**	**CBCT group**			**RVG group**		
	**Maxillary site (** ***n*** **= 12)**	**Mandibular site (** ***n*** **= 8)**	**Significance (** ***p*** **value)**	**Maxillary site (** ***n*** **= 12)**	**Mandibular site (** ***n*** **= 8)**	**Significance (** ***p*** **value)**
Mean DM_H_ (in mm)	0.0825	0.1225	0.256	0.65	0.436	0.354
Mean DM_EP_ (in mm)	0.361	0.436	0.570	0.505	0.871	0.149
Mean DM_T_ (in mm)	0.499	0.717	0.401	0.944	1.178	0.727
Mean DM_A_(in degrees)	4.416	5.125	0.683	4	9.5	0.099

DM_H_, deviation of height of mini-implant; DM_EP_, deviation of point of entry of mini-implant; DM_T_, deviation of tip of mini-implant; DM_A_, angular deviation of mini-implant from the ideal path.

In this study, three out of twenty mini-implants in the RVG group showed root contact. Of the three root contacts, one showed proximity to the second premolar and two showed proximity to the first molar. All the three mini-implants which contacted the root surface were placed in the mandibular arch and none of them showed any signs of failure during the loading period of 2 months.

One mini-implant in the RVG group was removed within the first week of loading due to increased mobility and, hence, was considered a failure. This failed mini-implant was not in contact with any of the neighboring roots.

## Discussion

The importance of accurate mini-implant positioning cannot be overemphasized. Accurate mini-implant positioning reduces problems such as loosening of the implant or invasion of the sinuses, periodontal ligament, or root surface and facilitates the use of proper force vectors during loading [22].

In this study, CBCT scan, to assess the possible sites for mini-implant placement, was done after preparatory orthodontic treatment unlike other studies [9–12] where CBCT scan was done prior to the treatment. Since the CBCT scan was not routinely done for all the patients for orthodontic diagnosis, hence, only after a confirmatory diagnosis patients were included in the study and exposed to CBCT. Secondly, any change in the root position and crown inclination during preparatory orthodontic treatment may interfere with the mini-implant positioning according to pretreatment CBCT. Also, taking CBCT after preparatory orthodontic treatment allowed the use of orthodontic arch wire as a standard reference line. Measurements done in relation to this standard reference can be used clinically without the need of expensive surgical guides/templates. Alveolar crest and cemento-enamel junction were not used as reference because these could not be seen and correlated clinically.

In the present study, the mini-implants in the RVG group were placed at a relatively lower height (i.e., closer to the arch wire) as compared to the ideal height determined, the mean (average) deviation being 0.565 mm. Considering the general guidelines for optimal sites for mini-implant placement as quoted by Fayed et al. [8] and Poggio et al [23] in which a range of different heights are given for mini-implant positioning, clinical relevance of the difference in height of mini-implants in this study is questionable. Considering the mean deviations in the mesiodistal direction namely the mean DM_EP_, DM_T_, and DM_A_, results showed a higher deviation in the RVG group, but the difference in the two groups was not statistically significant. And most of the mini-implants in both the groups were placed within the safe zone in the interradicular space.

Root contact was defined as the contact of mini-implant surface with the neighboring root in the CBCT images. Three out of twenty mini-implants in the RVG group showed root contact while others followed a path with varying amounts of linear and angular deviation from the ideal path without contacting the root surface. According to a study by Lee et al. [24], peri-root space might not be an absolute parameter for mini-implant stability as movement of about 0.5 mm was detected even in stable mini-implants under loading [25,26]. Fortunately, it is not necessarily detrimental even if the root is touched by the screw because almost complete root repair has been reported from an animal study [27]. The biologic nature and the osteodynamics around the orthodontic miniscrew under constant load need to be elucidated further in the future.

It is found that 15% of the mini-implants placed in the RVG group had root contact while none in the CBCT group had root contact. On careful evaluation of the postplacement CBCT, it was found that all the three mini-implants in the RVG group which contacted the root surface were placed in the mandibular interradicular space. The root contact may have occurred due to the presence of narrower interradicular spaces in the mandibular arches and reduced accessibility in the mandibular posterior region [8,23].

One mini-implant in the RVG group, which was removed within the first week of loading, was not in contact with any of the neighboring roots but was placed relatively closer to the arch wire. On retrospective evaluation, it was concluded that the reason for the failure of the mini-implant could be insufficient cortical bone thickness at the site of mini-implant placement which is thought to be a key determinant for initial stability of mini-implant [28–30].

The present study indicated no significant difference in deviation from the ideal placement of mini-implants between the right and left sides and between the maxillary and mandibular sites in each of the two groups. This shows that the operator was not biased towards any site during the mini-implant placement procedure.

From the above discussion, it can be stated that none of the two methods were able to guide the mini-implant accurately at the ideal position. However, most of the mini-implants in both the groups were placed in a safe zone without damaging the adjacent roots.

Fabrication of surgical stents, guides, and templates [9–12] is complicated, time-consuming, expensive, and required massive laboratory equipments and, hence, use of any of these latest technological advances for three-dimensional planning, and placement of mini-implants in the CBCT group was not considered.

Most clinicians use IOPA view of the region with a surgical guide in place to identify the site for mini-implant insertion. Hence, we used a grid in the RVG group in order to simulate the routine protocol used by most of the clinicians. However, no guide was used during insertion of the mini-implants. From the observations of our study, it can be concluded that the use of two-dimensional radiographs with a surgical guide is a more practical and cost-effective alternative to the three-dimensional planning with CBCT for routine mini-implant placement.

## Conclusions

The following can be concluded from this study.

- Although CBCT provides accurate three-dimensional visualization of the interradicular space, two-dimensional intraoral radiographs seem to provide sufficient information for mini-implant placement.
- It is recommended for clinicians to use two-dimensional radiographs with surgical guide for routine mini-implant placement.
- Considering the high cost and higher radiation dose as compared to two-dimensional radiographs, the routine use of CBCT is not recommended for orthodontic mini-implant placement. However, if mini-implant placement is difficult because of complex anatomy such as an expanded sinus or alveolar bone loss, the use of CBCT data for planning may be considered.

## Abbreviations

CBCT: Cone beam computed tomography
DM_A_: Angular deviation of the mini-implant from the ideal path
DM_EP_: Deviation of the point of entry of mini-implant
DM_H_: Deviation of height of mini-implant from the ideal height
DM_T_: Deviation of the tip of mini-implant
RVG: Radiovisiography
